# Characterization and phylogenetic analysis of the complete plastome of *Maclura tricuspidata* (Moraceae)

**DOI:** 10.1080/23802359.2021.1891986

**Published:** 2021-03-18

**Authors:** Xin-Xin Zhu, Rong Wang, Xue-Jie Zhang

**Affiliations:** Shandong Provincial Key Laboratory of Plant Stress Research, College of Life Sciences, Shandong Normal University, Ji’nan, Shandong, China

**Keywords:** *Maclura tricuspidata*, plastome, phylogenomics

## Abstract

The complete chloroplast genome (plastome) of *Maclura tricuspidate* Carriere, a thorny and deciduous tree, is determined. The plastome is 161,348 bp in length, consisting of a 89,364 bp large single-copy region, a 20,246 bp small single-copy region, and two 25,869 bp inverted repeat regions. The GC content of this plastome is 36.1%. A total of 112 unique genes are annotated for the plastome of *M. tricuspidata*, containing 78 protein coding genes (PCGs), 30 tRNAs, and four rRNAs. Phylogenetic analysis shows that *M. tricuspidata* is sister to the clade comprising Dorstenieae, Castilleae, and Ficeae.

*Maclura tricuspidate* Carriere is a member of the Moraceae family, mainly distributed in Korea and East Asia (Choi et al. 2020; Lee and Choi 2020; Park et al. 2020). It is a traditional medicinal plant given its biological activities (Park et al. 2020). It was found that *M. tricuspidata* fruit vinegar had anti-inflammatory effect *in vitro* (Choi et al. 2020). *M. tricuspidate*, a common food ingredient, has important medicinal and economic value. Several studies on the medicinal value of this plant have been reported (Nguyen et al. 2017; Choi et al. 2020; Kim and Park 2020; Lee and Choi 2020). However, the complete plastomes of *Maclura* have not been reported. Based on the previous studies of chloroplast gene *ndhF*, the position of *Maclura* was not resolved (Datwyler and Weiblen 2004). In this study, we present the plastome of *M. tricuspidata*, which would provide a fundamental genetic resource for studying this important species.

Fresh leaves of *M. tricuspidata* were collected from Shandong Forest Germplasm Resources Center (Shandong, China; 36°37′N, 117°9′E). Voucher specimen (SD334) was deposited at College of Life Sciences, Shandong Normal University. The total genomic DNA was used for library preparation and sequenced on an Illumina MiSeq instrument at Novogene (Beijing, China) with paired end reads of 150 bp. The total read number of the *M. tricuspidate* is 25,131,714. Then, we used Organelle Genome Assembler (OGA) pipeline (https://github.com/quxiaojian/OGA) to assemble plastome. Bowtie v2.3.4 was used for mapping raw reads, and Spades v3.7.1 was used to assemble mapped reads into contigs as described in Qu (2019). Plastome annotation was performed with Plastid Genome Annotator (PGA, https://github.com/quxiaojian/PGA) (Qu et al. 2019). Referring to previous published studies on the complete chloroplast genome (Wang et al. 2019; Guo et al. 2020), we use Geneious v9.1.4 for manual corrections (Kearse et al. 2012). A maximum-likelihood (ML) tree was reconstructed to determine the phylogenetic placement of *M. tricuspidata* using RAxML v8.2.10 (Stamatakis 2014), including tree robustness assessment using 1000 rapid bootstrap replicates with the GTRGAMMA substitution model, based on alignment of 78 shared protein-coding genes using MAFFT v7.313 (Katoh and Standley 2013).

The complete plastome of *M. tricuspidata* (GenBank accession number: MW244565) is 161,348 bp in length, consisting of a large single-copy region (89,364 bp), a small single-copy region (20,246 bp), and a pair of inverted repeats regions (25,869 bp). The GC content of this plastome is 36.1%. In total, 112 unique genes are encoded, including 78 PCGs, 30 tRNAs, and four rRNAs. Phylogenetic analysis shows that *M. tricuspidata* is sister to the clade comprising Dorstenieae, Castilleae, and Ficeae (Figure 1).

**Figure 1.**
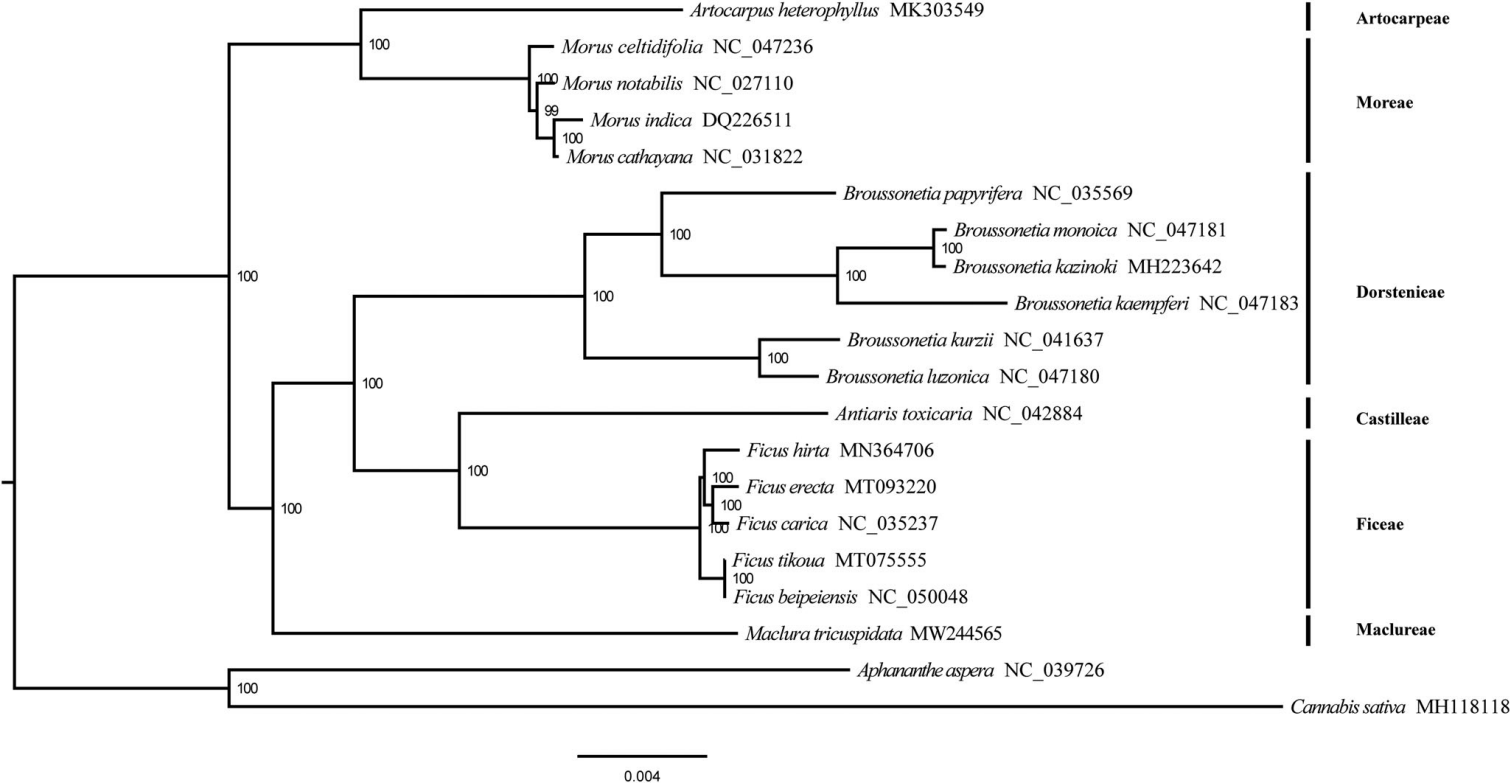
The maximum likelihood (ML) tree is reconstructed by 78 plastome genes. *Aphananthe aspera* and *Cannabis sativa* are used as out-group. Bootstrap support values are indicated at the node of the ML tree.

## Data Availability

The data that support the findings of this study are openly available in GenBank of NCBI at https://www.ncbi.nlm.nih.gov, reference number MW244565. The data that new raw obtained at this study are available in the NCBI under accession number of SRR13052618.
